# FOXD2-AS1 promotes malignant cell behavior in oral squamous cell carcinoma via the miR-378 g/CRABP2 axis

**DOI:** 10.1186/s12903-024-04388-2

**Published:** 2024-05-28

**Authors:** Shaoyong Guo, Bixia Huang, Zhisong You, Zhenzhi Luo, Da Xu, Jieru Zhang, Jialin Lin

**Affiliations:** 1 Department of Stomatology, The First Hospital of Putian City, 449 Nanmen West Road, Chengxiang District, Putian City, Putian 351100 China; 2 Department of Neurology, The Affiliated Hospital of Putian University, Putian, 351100 China

**Keywords:** ceRNA network, Functional experiments, Invasion, lncRNA, Migration

## Abstract

**Background:**

Oral squamous cell cancer (OSCC) is a prevalent malignancy in oral cavity, accounting for nearly 90% of oral malignancies. It ranks sixth among the most common types of cancer worldwide and is responsible for approximately 145,000 deaths each year. It is widely accepted that noncoding RNAs participate cancer development in competitive regulatory interaction, knowing as competing endogenous RNA (ceRNA) network, whereby long non-coding RNA (lncRNA) function as decoys of microRNAs to regulate gene expression. LncRNA FOXD2-AS1 was reported to exert an oncogenic role in OSCC. Nevertheless, the ceRNA network mediated by FOXD2-AS1 was not investigated yet. This study aimed to explore the effect of FOXD2-AS1 on OSCC cell process and the underlying ceRNA mechanism.

**Methods:**

FOXD2-AS1 expression in OSCC cells were determined via reverse transcription and quantitative polymerase chain reaction. Short hairpin RNA targeting FOXD2-AS1 was transfected into OSCC cells to silence FOXD2-AS1 expression. Then, loss-of-function experiments (*n* = 3 each assay) were performed to measure cell proliferation, apoptosis, migration, and invasion using colony formation, TdT-mediated dUTP Nick-End Labeling, wound healing and Transwell assays, respectively. RNA binding relation was verified by RNA immunoprecipitation and luciferase reporter assays. Rescue experiments were designed to validate whether FOXD2-AS1 affects cell behavior via the gene cellular retinoic acid binding protein 2 (CRABP2). Statistics were processed by GraphPad Prism 6.0 Software and SPSS software.

**Results:**

FOXD2-AS1 was significantly upregulated in Cal27 and SCC9 cells (6.8 and 6.4 folds). In response to FOXD2-AS1 knockout, OSCC cell proliferation, migration and invasion were suppressed (approximately 50% decrease) while OSCC cell apoptosis was enhanced (more than two-fold increase). FOXD2-AS1 interacted with miR-378 g to alter CRABP2 expression. CRABP2 upregulation partly rescued (*p < 0.05, **p < 0.01, ***p < 0.001) the inhibitory impact of FOXD2-AS1 depletion on malignant characteristics of OSCC cells.

**Conclusion:**

FOXD2-AS1 enhances OSCC malignant cell behaviors by interacting with miR-378 g to regulate CRABP2 expression.

**Supplementary Information:**

The online version contains supplementary material available at 10.1186/s12903-024-04388-2.

## Introduction

Oral Squamous Cell Carcinoma (OSCC) is a common type of head and neck malignancy and occurs in the oral mucosa [1]. Each year, there are over 350,000 newly confirmed cases and approximately 145,000 deaths worldwide [2]. As reported, exacerbated inflammation and deficient nutrition are common factors causing this cancer [3]. In some hospitals, the department of oral and maxillofacial (OMF) surgery was set up to treat tumors of oral, face and jaws [4]. Many OMF tumors were cured with wide or radical surgery. Anatomy, reconstructions, and long-surgical procedures are necessary for management of advanced head and neck cancer and/or maxillofacial trauma [5]. As previously reported, reconstruction of facial defect using deltopectoral flap was validated to be feasible [5]. Other cases of OMF tumors achieved temporary control by removing infection and the source of pain so as to keep masticatory function and improve the life quality of patients [6]. For example, the use of diode laser ablation produced outstanding esthetic and functional outcomes during a 6-month follow-up period [7]. Another study further validated that the clinical application of the diode (980 nm) laser in maxillofacial surgery is safe, acceptable, practical, and easy to be used [8]. Currently, great improvements have been made in radiotherapy, chemotherapy, and multidisciplinary comprehensive sequence therapy for OSCC treatment [9]. However, there is a long way to go to improve the prognosis of patients with OSCC and their quality of life at the same time. In OSCC, the organ that is important for talking and chewing food may be destructed. Surgery has the least negative impact on the quality of life; however, it is necessary for patients to receive other treatments such as chemotherapy or radiotherapy if the surgeon has difficulty accessing the primary lesion. As known, combined therapy of chemotherapy and radiotherapy exerts the most negative effect on the quality of life because of dry mouth side effects induced by these treatment, thereby influencing physical and mental state of patients [10]. The five-year survival rate for OSCC is only 50% and is not optimal [11]. With the rapid development of nanotechnology, nanodrug delivery systems has been widely utilized in targeted therapy for OSCC. Active and passive targeted therapies are promising options for clinical transformation and application of OSCC [12]. Therefore, further exploring the molecular mechanisms underlying OSCC tumorigenesis is urgent.

Oncogenes induces the initiation and progression of OSCC, and most of them are protein-coding genes [13]. Nevertheless, protein-coding genes only take up 2% of human genome, while non-coding RNAs (ncRNAs) account for 70% [14]. The ncRNAs consist of long ncRNAs (lncRNA) and small ncRNAs [15]. LncRNA plays a critical role in regulating gene expression [16]. Molecular mechanisms of lncRNA in OSCC include epigenetic regulation, transcriptional regulation, and post-transcriptional regulation [17]. The present study explored the function of lncRNA as a post-transcriptional regulator by sponging microRNA (miRNA) and regulating mRNA translation. To be specific, lncRNA interacts with miRNA, behaving as a competing endogenous RNA (ceRNA) and counteracting the inhibitory effect of the miRNA on mRNA expression [18]. Regarding their biological roles in various types human cancers including OSCC, lncRNAs have been validated to perform modulatory functions that affecting cell biological behaviors, immune response, and transformed phenotype in cells [17]. Cancer cell biological behaviors include cell proliferation, migration, invasion, drug resistance and apoptosis [19–21]. In addition, lncRNA is distributed abundantly in body fluids including urine, blood, saliva, and exosomes. Thus, lncRNA can be regarded as a type of non-invasive biomarker [22]. LncRNAs with aberrant expression can be identified by RNA sequencing and lncRNA-microarray profiling. For example, Ganesan Arunkumar et al. performed RNA sequencing analysis of oral cancer tissues in India and found that 11 dysregulated lncRNA in OSCC are all strongly related to tobacco chewing or have smoking history [23]. Therefore, identifying more biomarkers in OSCC and investigating their functions are meaningful for future studies. However, the identified lncRNAs at present are limited and their mechanisms require further exploration. A previous study has revealed that FOXD2-AS1 overexpression indicates unfavorable outcomes in patients with OSCC [24]. Bioinformatics analysis shows that FOXD2-AS1 is dysregulated in head and neck squamous cell carcinoma (HNSC) tissues. However, the regulatory mechanism of FOXD2-AS1 in OSCC development is still unclear. Therefore, FOXD2-AS1 was identified for exploration in this study. Previously, FOXD2-AS1 dysregulation was shown to affect various biological processes in different cancers. FOXD2-AS1 aggravates lung cancer through the Wnt/β-catenin signaling [25], promotes gastric cancer via epigenetically knockdown Ephrin B3 [26], and contributes to colorectal cancer progression [27].

We herein analyzed the molecular mechanism of FOXD2-AS1 in OSCC and aimed to explore whether FOXD2-AS1 can promote OSCC cell growth and metastasis by sponging miR-378 g and restraining the suppressive effect of miR-378 g on CRABP2 expression. The downstream factors miR-378 g and CRABP2 were selected based on bioinformatics analysis. The study might provide a novel target and a promising strategy for diagnosis and targeted therapy in OSCC.

## Material & method

### Cell culture

Human OSCC cell lines, including Cal27 (#YS657C), SCC9 (#YS336C), HSC-3 (#YS1645C), and human oral keratinocytes (HOK; #YS1199C) were obtained from Yaji Biological (Shanghai, China). Dulbecco’s modified Eagle’s medium (DMEM; 45 mL; Procell, Wuhan, China) added with 10% fetal bovine saline (5 mL), 1% glutamine, and 1% penicillin/streptomycin (500 µL) was prepared for cell incubation. The culture medium was changed every 2 days. Cells were incubated in a humidified atmosphere containing 5% CO_2_ at 37℃.

### Cell transfection

Cell transfection was performed to overexpress or knockdown RNA expression in OSCC cells through transfection of specific plasmids, and the procedure was prepared for subsequent functional experiments or regulatory relationship exploration. The short hairpin RNAs (shRNAs; Genechem, Shanghai, China) against FOXD2-AS1 (sh-FOXD2-AS1#1/2) and negative control (sh-NC) were used to silence FOXD2-AS1 expression. MiR-378 g mimic and NC mimic were synthesized by GenePharma (Shanghai, China) and used for miR-378 g overexpression. CRABP2 cDNA was cloned into pcDNA3.1 vectors to overexpress CRABP2, and empty pcDNA3.1 vectors were utilized as the negative control. The concentrations of shRNAs, miR-378 g mimic/NC mimic, and pcDNA3.1 vectors are 40 nM, 35 nM, and 10 nM, respectively. Cal27 and SCC9 cells were seeded in six-well plates. Lipofectamine 2000 (Ziker, Shenzhen, China) was used following the manufacturer’s protocols to perform 48 h of cell transfection.

### Reverse transcription quantitative polymerase chain reaction (RT-qPCR)

Expression levels of FOXD2-AS1, miR-378 g, and mRNAs were measured by RT-qPCR. TRIzol reagents kits (Invitrogen, Carlsbad, USA) were used to extract total RNAs from OSCC cells according to the manufacturer’s protocols. RNA samples were then reverse transcribed into cDNAs using PrimeScript reverse transcription reagent kits (BioPike, Beijing, China) in line with product instruction. PCR was conducted on the ABI 7500 system with SYBR Green Premix PCR Master Mix (Sigma-Aldrich, St-Louis, MO, USA) to prepare PCR reaction systems as instructed. PCR was performed at 95 °C for 10 min, followed by 40 cycles of 95 °C for 30 s and 60 °C for 1 min. The 2^−△△Ct^ method was used to measure the relative RNA expression. U6 was utilized as the internal reference of miR-378 g, while FOXD2-AS1 and CRABP2 levels were normalized to GAPDH. The primer sequences of CRABP2 were obtained from GETprime [28] and sequences of FOXD2-AS1 and miR-378 g were designed by Thermo Fisher Scientific (Waltham, MA, USA). All primer sequences were listed in Table 1.

**Table 1:** Sequences of primers used for reverse transcription-quantitative PCR

Gene	Sequence (5’→3’)
FOXD2-AS1 forward	GCGAAGAGTACGTTGCTATCA
FOXD2-AS1 reverse	GCGTGCAATCGTTCCGCTGTG
miR-378 g forward	ACACTCCAGCTGGGGAAGACTGAGGTTC
miR-378 g reverse	CTCAACTGGTGTCGTGGAGTCGGCAATTCAGTTGAGAGCCCAGT
CRABP2 forward	CACCACAGAGATTAACTTCAAGG
CRABP2 reverse	TTCACCAGGCTCTTACAGG
GAPDH forward	ACCACAGTCCATGCCATCAC
GAPDH reverse	TCCACCACCCTGTTGCTGTA
U6 forward	CTCGCTTCGGCAGCACA
U6 reverse	AACGCTTCACGAATTTGCGT

### Subcellular fractionation assay

The distribution of FOXD2-AS1 in Cal27 and SCC9 cells was determined by the subcellular fractionation assay. The nuclear and cytoplasmic RNA was extracted using the PARIS™ kit (Yu Biotech, Hangzhou, China). In brief, cells were resuspended in 300-µl ice-cold cell fractionation buffer. Then the mixture was incubated on ice for 5–10 min. The supernatant was skimmed after centrifugation at 500 ×g for 3 min, and the nuclear pellets was left. Then, 400-µl ice-cold cell disruption buffer and 400-µl 2×lysis/binding solution were added to the nuclei. Finally, elution solution was prepared for RNA elution at 95℃. The percentage of FOXD2-AS1, GAPDH, and U6 in cytoplasmic and nuclear parts was quantified by RT-qPCR.

### Cell viability detection

The viability of OSCC cells was assessed using Cell Counting Kit-8 (CCK-8) reagents (Sigma Aldrich, St. Louis, USA). Cal27 and SCC9 cells were inoculated into 96-well plates (1 × 10^3^ cells/ well). Then 10-µl CCK-8 reagents were added into each well at 0 h, 24 h, 48 h and 72 h. After 2 h-incubation, the optical density (OD) was determined at the wavelength of 450 nm using F500 microplate reader (Thermo Fisher Scientific).

### Colony formation assay

After abovementioned transfection, OSCC cell proliferation was evaluated using colony formation assay. Cal27 and SCC9 cells were grown in the culture plate (3 × 10^3^/100 µl) in an incubator for another 24 h at 37 °C containing 5% CO_2_. Every two days, the culture medium was changed. After 14 days, the incubation was terminated. Cells were purified twice with phosphate buffered saline (PBS) (Suntip, Guangdong, China) after removing the solution. Colonies were fixed with 5% paraformaldehyde (Macklin, Shanghai, China) and then stained with 1 ml of 0.1% crystal violet solution (Le Heng Technology, Shenyang, China). After PBS-washing, the colony number was counted using a gel documentation system (Thermo Fisher Scientific).

### Wound healing assay

Wound healing assays were conducted to measure OSCC cell migration in response to FOXD2-AS1 knockdown or CRABP2 overexpression. Cal27 and SCC9 cells with indicated treatment were grown in 6-well plates until full confluence. A homogenous wound was drawn using a sterile plastic micropipette tip. Then, cells were incubated in serum-free DMEM medium (Thermo Fisher Scientific) for 24 h. The wound movement was observed and imaged at 0 h and 24 h using a light microscope (Olympus Corporation, Tokyo, Japan).

### Transwell assay

After indicated transfection, OSCC cell invasion was measured by Transwell assays using Corning Costar transwell chambers (Corning incorporated, Corning, USA) precoated with Matrigel (BD Biosciences, San Jose, USA). OSCC cells (2 × 10^4^) were cultured into single-cell suspension in serum-free medium. Then the mixture was added to the upper chamber. Complete medium containing 15% fetal bovine saline was added to the lower chamber. After 24 h, cells in the upper chamber were removed, and cells invaded to the lower membrane were fixed with 4% paraformaldehyde and stained with 0.25% crystal violet solution. Finally, we selected five fields at random to count the number of invaded cells using an inverted microscope (×200) (Thermo Fisher Scientific).

### RNA immunoprecipitation (RIP) assay

RIP assays were carried out using the EZ-Magna RIP kit (Millipore, Billerica, MA). To explore the relationship between FOXD2-AS1 and miR-378 g, Cal27 and SCC9 cells transfected with NC mimics and miR-378 g mimics were lysed in lysis buffer containing protease inhibitor cocktail and RNase inhibitor. After lysis, RIP buffer containing magnetic beads coated with Ago2 antibodies (Millipore) was prepared for cell incubation. After 2 h of incubation, the coprecipitated RNAs were washed out from the beads and measured by RT-qPCR. IgG served as a negative control. To explore the relationship among FOXD2-AS1, miR-378 g, and CRABP2, the same procedures were conducted except the transfection of mimics before cell lysis.

### Luciferase reporter assay

Luciferase reporter assays were carried out to validate the binding between miR-378 g and FOXD2-AS1 (or CRABP2) in Cal27 and SCC9 cells. Wide type (Wt) or mutant (Mut) sequence of CRABP2 3′-untranslated region (3′-UTR) was cloned into the firefly luciferase gene reporter vector pmirGLO (Promega, Madison, USA). The pmirGLO-CRABP2-Wt or pmirGLO-CRABP2-Mut was co-transfected with miR-378 g mimics or NC mimics into Cal27 and SCC9 cells. Similarly, the pmirGLO-miR-378 g-Wt or pmirGLO-miR-378 g-Mut was established and then co-transfected with sh-NC or sh-FOXD2-AS1#1 into OSCC cells. The dual-luciferase reporter assay system kit (Promega) and Modulus single-tube multimode reader (Promega) were used to detect the luciferase activities. The relative luciferase activity was normalized to the content of the Renilla.

### TdT-mediated dUTP Nick-End labeling (TUNEL)

Cal27 and SCC9 cell apoptosis after transfection of plasmids was measured by TUNEL assays. Cells were fixed with 4% paraformaldehyde in PBS which contained 0.12 mM sucrose for 15 min. Apoptosis was determined using the terminal doxynucleotidy1 TUNEL apoptosis kit (Sangon Biotech) in line with the manufacturer’s recommendations. The apoptotic cells were labelled, and the percentage of TUNEL-positive cells was observed and counted using a fluorescence microscope (Carl Zeiss, Oberkochen, Germany).

### Statistical analysis

Statistics were analyzed using GraphPad Prism 6.0 Software (GraphPad Inc, San Diego, USA) and Statistical Product and Service Solutions (SPSS) software (Chicago, USA). Student’s *t*-test was utilized to compare significance between two groups, and one-way analysis of variance (ANOVA) followed by Tukey’s *post hoc* analysis were used to compare differences among multiple groups. Differences were defined as statistically significant when *p* < 0.05.

## Results

### High FOXD2-AS1 level is observed in OSCC cells

**Fig. 1:**
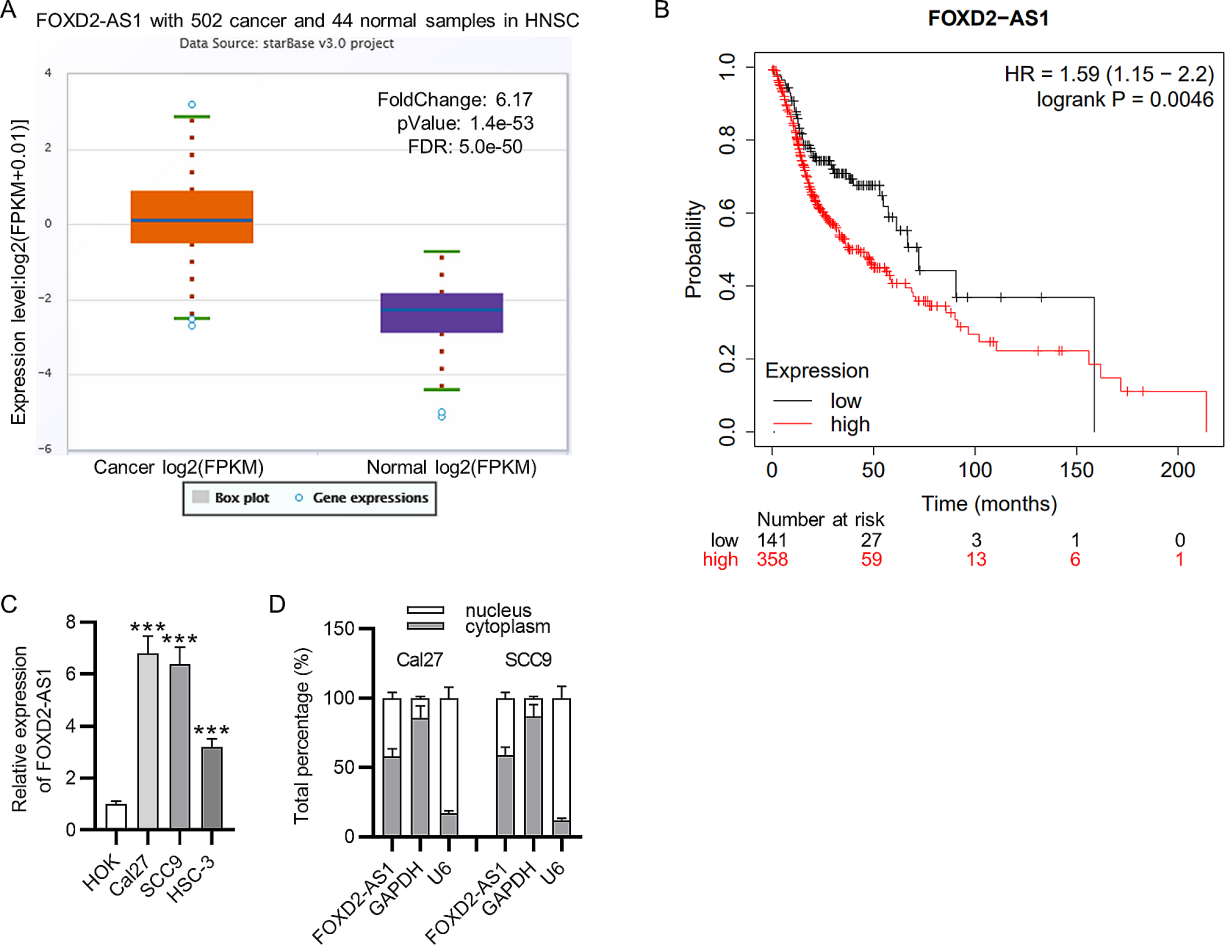
High FOXD2-AS1 level is observed in OSCC cells. **(A)** FOXD2-AS1 expression in HNSC tissues was analyzed by ENCORI (https://rnasysu.com/encori/index.php). **(B)** Correlations between FOXD2-AS1 expression and prognosis of patients with OSCC were analyzed by Kaplan Meier plotter (http://kmplot.com/analysis/index.php?p=service). **(C)** FOXD2-AS1 expression in OSCC cells and human oral keratinocytes was measured by RT-qPCR. **(D)** Subcellular fractionation assays was performed to determine the distribution of FOXD2-AS1 in OSCC cells. ^***^p < 0.001

A previous article reported that FOXD2-AS1 correlates to unfavorable outcomes in patients with OSCC [24]. Bioinformatics analyses further verify the significance of FOXD2-AS1 in head and neck squamous cell carcinoma (HNSC). ENCORI is a bioinformatics tool for RNA interactomes and has pan-cancer differential expression analysis of genes across 32 types of cancer [29] and is used for prediction of FOXD2-AS1 expression with default settings. It reveals the upregulation of FOXD2-AS1 in HNSC tissues (*n* = 502) (Fig. 1A). Additionally, a higher FOXD2-AS1 level indicates a poor overall survival rate in patients with HNSC (Fig. 1B). FOXD2-AS1 level was elevated in OSCC cells (Cal27, SCC9, HSC-3) compared to that in HOK cells (Fig. 1C, *p* < 0.001, *t*-test). To determine the main distribution of FOXD2-AS1 in OSCC cells, cytoplasmic and nuclear parts in OSCC cells were isolated and FOXD2-AS1 level in two parts was measured by RT-qPCR. GAPDH was the internal reference for the cytoplasmic part while U6 was the internal control for the nuclear part. As shown by Fig. 1D, the percentage of FOXD2-AS1 is much higher in cytoplasm than that in nucleus. Thus, it can be concluded that FOXD2-AS1 is mainly localized in cytoplasm, indicating that FOXD2-AS1 could participate in OSCC progression post-transcriptionally.

### FOXD2-AS1 knockdown inhibits cellular processes in OSCC

**Fig. 2:**
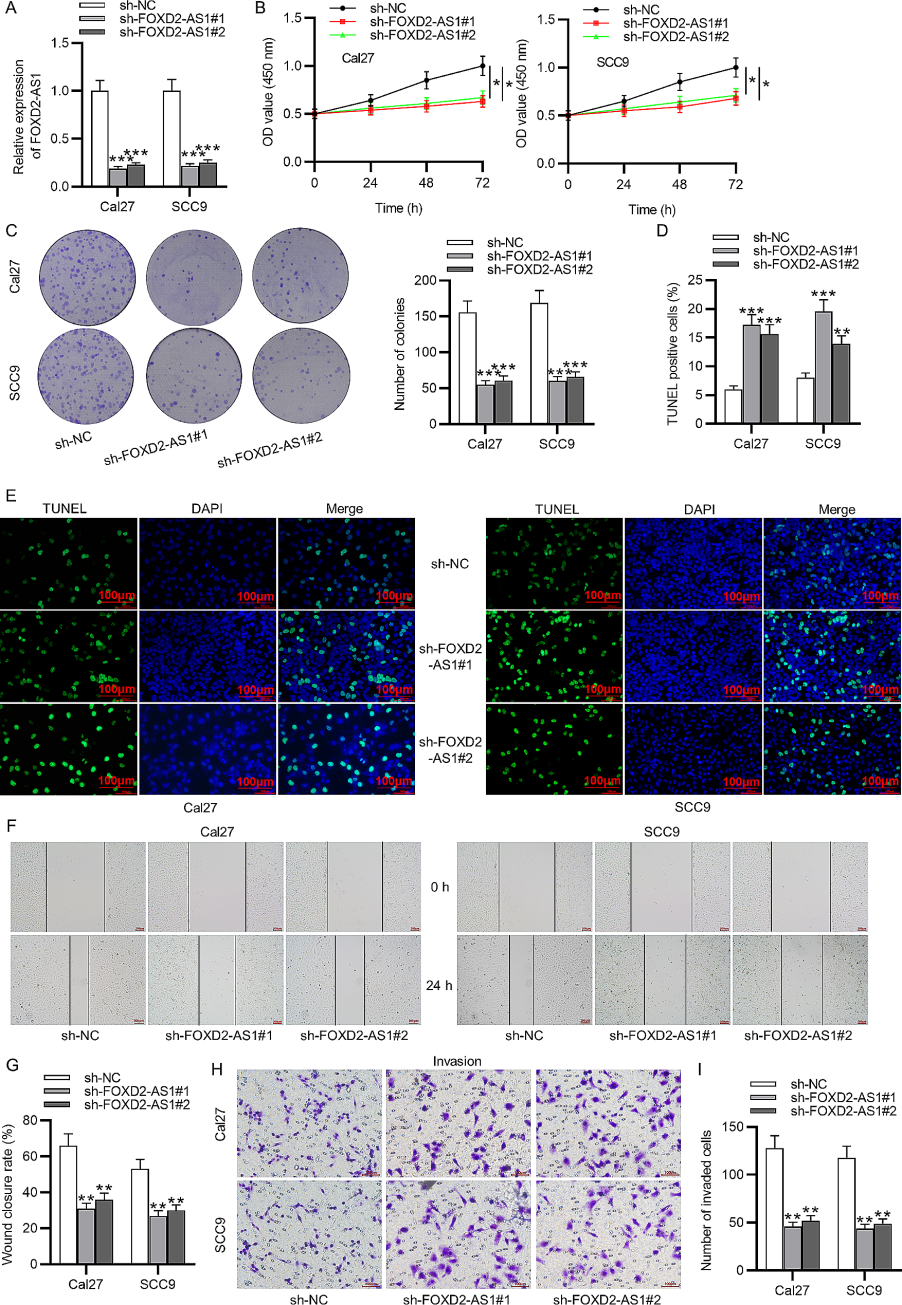
FOXD2-AS1 knockdown suppresses cellular processes in OSCC. **(A)** FOXD2-AS1 expression was knocked down by transfection of sh-FOXD2-AS1#1/2 into OSCC cells. **(B-C)** CCK-8 and colony formation assays were carried out to assess cell viability and proliferation. **(D-E)** TUNEL assays were conducted to evaluate cell apoptosis. **(F-I)** Wound healing assays and Transwell assays were performed for measurement of cell migration and invasion. ^*^p < 0.05, ^**^p < 0.01, ^***^p < 0.001

Considering high FOXD2-AS1 level in OSCC cells, we effectively knocked down FOXD2-AS1 level by transfecting sh-FOXD2-AS1#1/2 into Cal27 and SCC9 cells for 48 h using Lipofectamine 2000. As expected, FOXD2-AS1 expression was markedly lowered in OSCC cells with the transfection of sh-FOXD2-AS1#1/2 compared to its expression in sh-NC group (Fig. 2A, ***p < 0.001). Then, loss-of-functional experiments were carried out, and each assay was repeated three times. According to CCK-8 and colony formation assays, FOXD2-AS1 silencing significantly reduced the optical density values and the number of cell colonies, indicating the suppression of cell viability and proliferation in response to FOXD2-AS1 knockdown (Fig. 2B-C, *p < 0.01, **p < 0.01, ***p < 0.001, *t*-test). FOXD2-AS1 silencing led to an increase in the proportion of TUNEL-positive cells, implying the enhancing effect of FOXD2-AS1 on OSCC cell apoptosis (Fig. 2D-E, **p < 0.01, ***p < 0.001, *t*-test). Cell migration and invasion were suppressed after FOXD2-AS1 knockdown, as wound healing assays and Transwell assays revealed (Fig. 2F and I, **p < 0.01, *t*-test). Collectively, FOXD2-AS1 knockdown inhibits the malignant phenotypes of OSCC cells.

### FOXD2-AS1 binds to miR-378 g

**Fig. 3:**
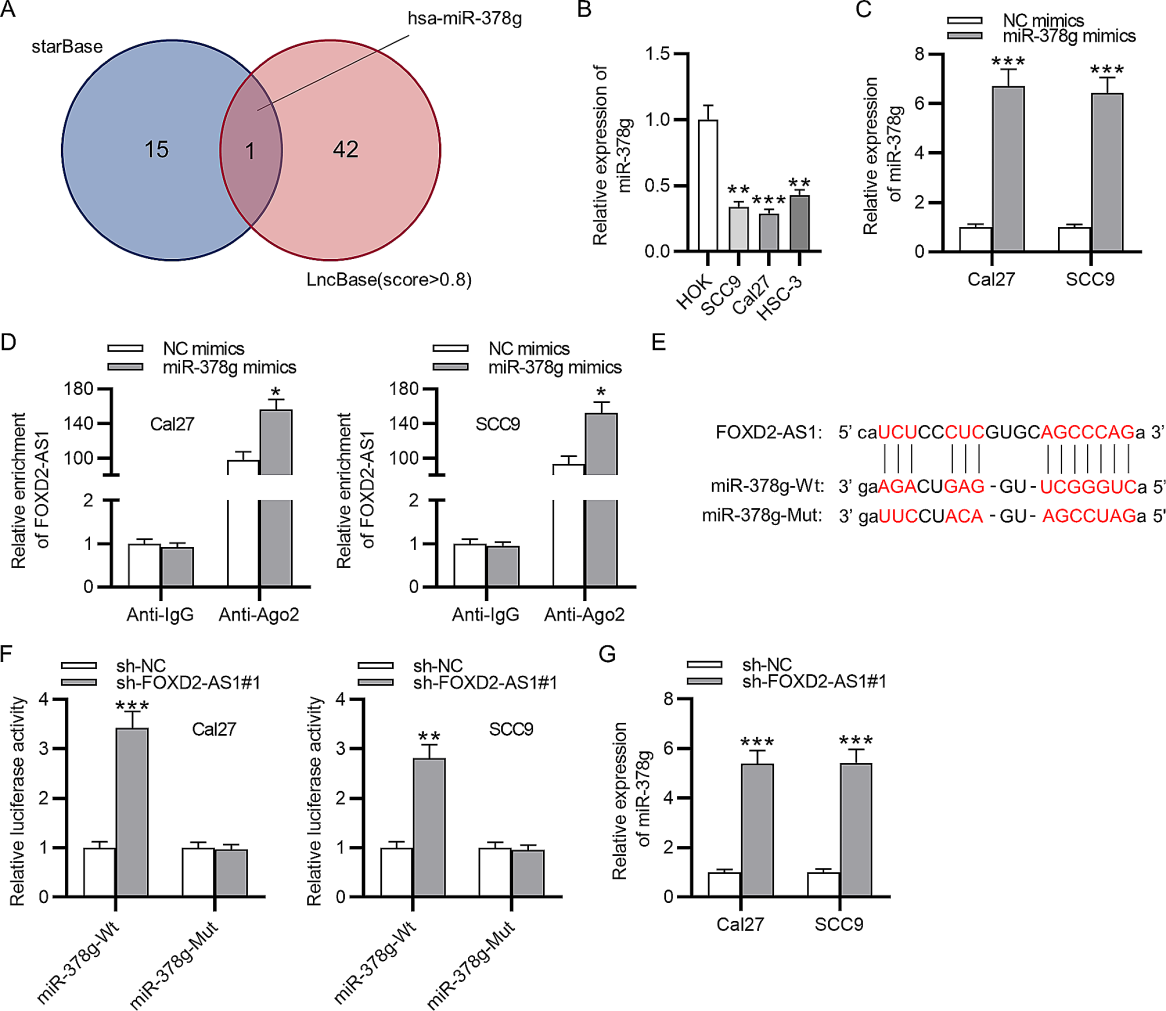
miR-378 g has binding site for FOXD2-AS1. **(A)** ENCORI were used to predict miRNAs that can interact with FOXD2-AS1. **(B)** RT-qPCR analysis of miR-378 g expression in OSCC cells. **(C)** The overexpression efficiency of miR-378 g mimics was measured by RT-qPCR. **(D)** The binding capacity of miR-378 g for FOXD2-AS1 was determined by RIP assays. **(E)** The binding site between FOXD2-AS1 and miR-378 g. **(F)** Luciferase reporter assays were conducted to verify the interaction between FOXD2-AS1 and miR-378 g. **(G)** RT-qPCR analysis of miR-378 g level after FOXD2-AS1 knockdown. ^*^p < 0.05, ^**^p < 0.01, ^***^p < 0.001

The mechanisms by which FOXD2-AS1 affects OSCC cell behavior was investigated. MiR-378 g was identified using the function of miRNA-lncRNA prediction (default setting) in ENCORI (previously named starBase) and another tool of LncBase with the screening criterion of score > 0.8 (Fig. 3A). RT-qPCR showed the downregulation of miR-378 g in OSCC cells (Fig. 3B, **p < 0.01, ***p < 0.001, *t*-test). Thus, we effectively overexpressed miR-378 g with transfection of miR-378 g mimics for 48 h using Lipofectamine 2000 (Fig. 3C, ***p < 0.001, *t*-test). Furthermore, RIP assays were performed to explore whether FOXD2-AS1 and miR-378 g were in the same RNA-induced silencing complex. The miR-378 g mimics and NC mimics were transfected into Cal27 and SCC9 cells and then cells were lysed and incubated with RIP buffer containing magnetic beads precoated with Ago2 antibody. The anti-IgG group was set as the control. RT-qPCR was used to quantify FOXD2-AS1 expression in different groups. The results demonstrated that FOXD2-AS1 was highly enriched in anti-Ago2 group compared with that in anti-IgG group, and miR-378 g mimics in the anti-Ago2 group further increased FOXD2-AS1 enrichment in Cal27 and SCC9 cells, suggesting that FOXD2-AS1 and miR-378 g were in RNA-induced silencing complex (Fig. 3D, *p < 0.05, *t*-test). The binding site of miR-378 g for FOXD2-AS1 was obtained from ENCORI (Fig. 3E). Moreover, luciferase reporter assays were conducted for verification of the interaction between FOXD2-AS1 and miR-378 g. The pmirGLO vectors containing the wt or mut sequences of miR-378 g were co-transfected with sh-FOXD2-AS1#1/2 into Cal27 and SCC9 cells for 48 h. Firefly activities in various groups were then assessed with normalization to Renilla activity. It was revealed that sh-FOXD2-AS1#1/2 increased the luciferase activity of vectors containing miR-378 g-Wt while the activity of miR-378 g-Mut had no significant changes in response to miR-378 g overexpression (Fig. 3F, **p < 0.01, ***p < 0.001, *t*-test). Finally, according to the result of RT-qPCR, FOXD2-AS1 knockdown induced high expression of miR-378 g, indicating their negative expression correlation in OSCC cells (Fig. 3G, ***p < 0.001, *t*-test). Taken together, these data demonstrated that FOXD2-AS1 directly interacted with miR-378 g.

### MiR-378 g targets CRABP2 in OSCC cells

**Fig. 4:**
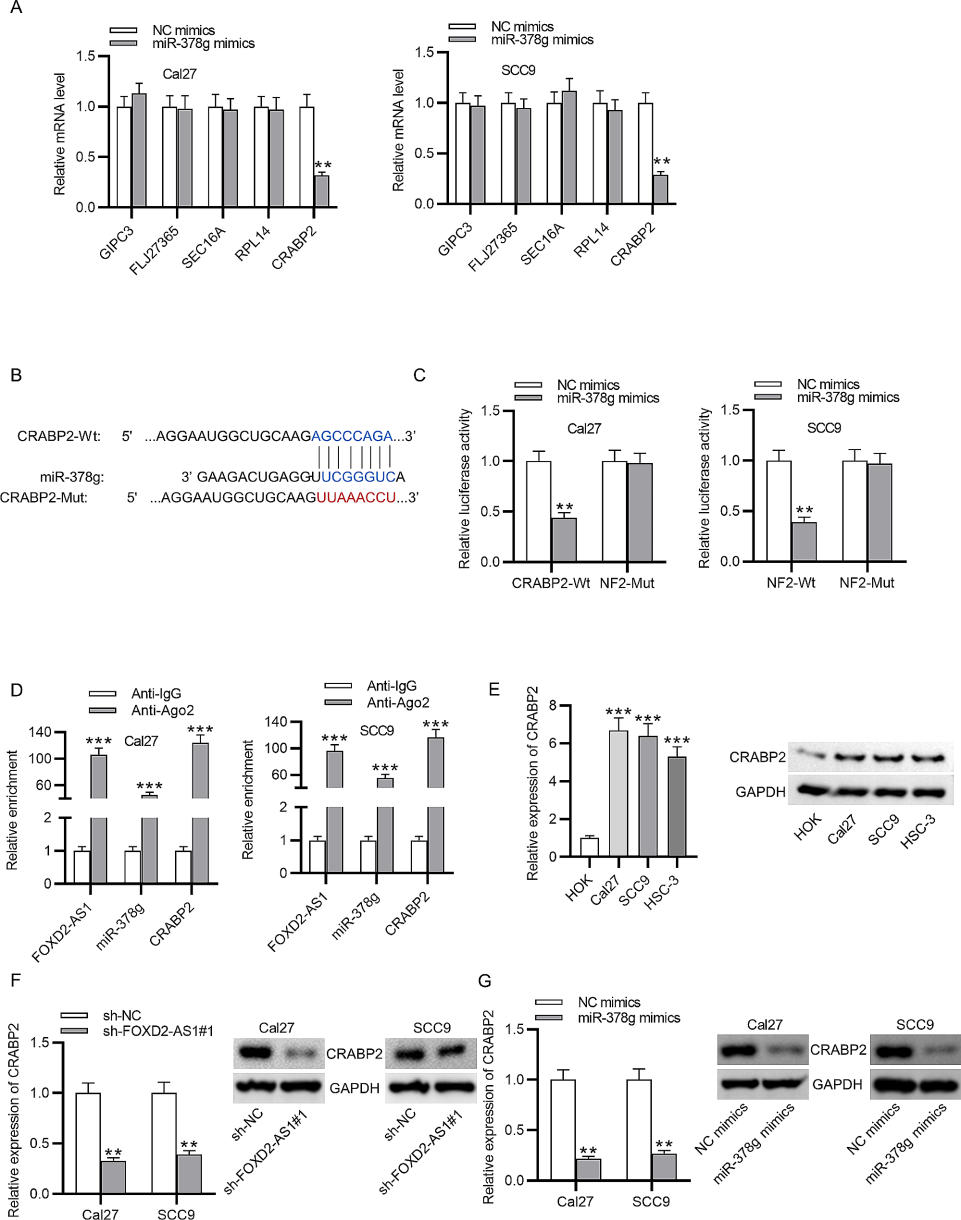
FOXD2-AS1 upregulates CRABP2 expression via binding with miR-378 g. **(A)** The bioinformatics tool TargetScan was used to identify potential targets of miR-378 g. **(B)** The binding area between miR-378 g and CRABP2. **(C-D)** Luciferase reporter assays and RIP assays were conducted to confirm the binding between CRABP2 and miR-378 g. **(E)** RT-qPCR and western blotting of CRABP2 mRNA and protein levels in OSCC cells. **(F-G)** RT-qPCR and western blot analyses of CRABP2 mRNA and protein levels after FOXD2-AS1 knockdown or miR-378 g upregulation. ^**^p < 0.01, ^***^p < 0.001

The target genes regulated by miR-378 g were predicted using TargetScan with default settings, and the first five genes with high total context score were preliminary identified. Then, PCR analysis was performed to measure the expression of these five genes in OSCC cells in response to miR-378 g overexpression. As illustrated by Fig. 4A, CRABP2 expression is markedly reduced in OSCC cells amplifying miR-378 g expression (**p < 0.01, *t*-test), while the expression levels of other four genes were not significantly altered between miR-378 g mimics group and NC mimics group. Thus, CRABP2 was identified for subsequent experiments since only its expression was negatively correlated with miR-378 g in OSCC cells. The binding area between miR-378 g and CRABP2 was obtained from TargetScan, and the mutant sequence of CRABP2 is also provided in Fig. 4B. Then luciferase reporter assays were conducted, and the results demonstrated the decreased luciferase activity of CRABP2-Wt in the context of miR-378 g overexpression (**p < 0.01, *t*-test), whereas there was no obvious change of luciferase activity in CRABP2-Mut after miR-378 g interference (Fig. 4C). RIP assays revealed that FOXD2-AS1, miR-378 g, and CRABP2 were highly expressed in Ago2 antibody group compared to their levels in IgG group (Fig. 4D, ***p < 0.01, *t*-test). Moreover, CRABP2 mRNA and protein levels were highly expressed in OSCC cells (Fig. 4E, ***p < 0.01, *t*-test). FOXD2-AS1 knockdown prominently suppressed the mRNA and protein levels of CRABP2, and miR-378 g upregulation exerted the same effect on CRABP2 levels (Fig. 4F-G, **p < 0.01, *t*-test). Thus, above results strongly verify that FOXD2-AS1 binds to miR-378 g to increase CRABP2 expression.

### CRABP2 restores the inhibited malignant phenotypes of OSCC cells mediated by sh-FOXD2-AS1

**Fig. 5:**
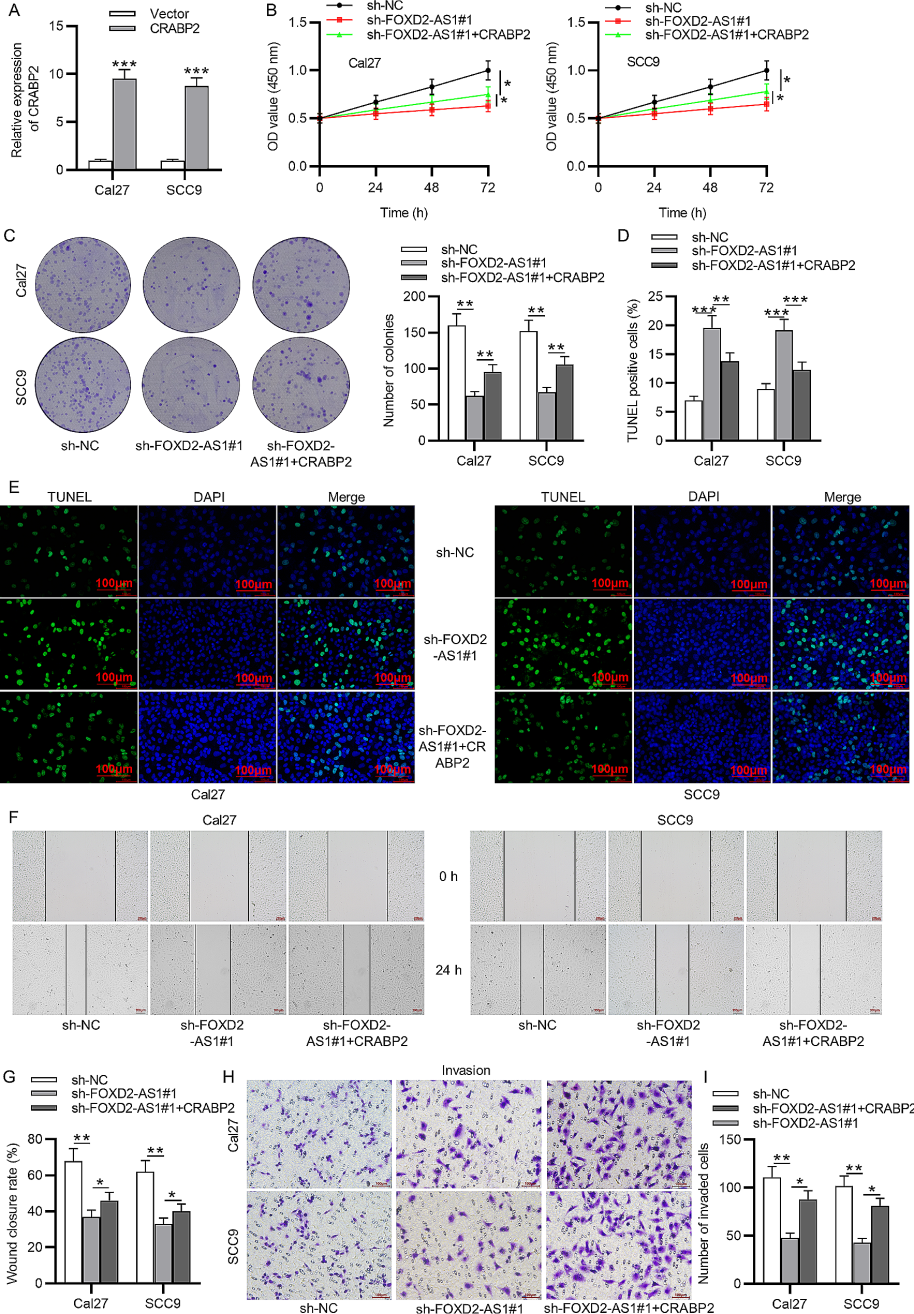
FOXD2-AS1 enhances malignant phenotypes of OSCC cells by regulating CRABP2 expression. **(A)** The overexpression efficiency of pcDNA3.1-CRABP2 was assessed by RT-qPCR. **(B)** CCK-8 assays were conducted to detect cell activity after transfection with sh-NC or sh-FOXD2-AS1#1 or sh-FOXD2-AS1#1 + CRABP2. **(C)** Colony formation assays to evaluate cell proliferation. **(D-E)** TUNEL assays to measure cell apoptosis. **(F-G)** Wound healing assays to determine cell migration. **(H-I)** Transwell assays to assess cell invasion. ^*^p < 0.05, ^**^p < 0.01, ^***^p < 0.001

Then we effectively increased CRABP2 level in Cal27 and SCC9 cells through transfection of pcDNA3.1-CRABP2 vectors into Cal27 and SCC9 cells for 48 h using Lipofectamine 2000 (Fig. 5A, ***p < 0.001, *t*-test). As CCK-8 assays revealed, CRABP2 overexpression counteracted the reduction of cell viability induced by FOXD2-AS1 downregulation (Fig. 5B, *p < 0.05, ANOVA). The inhibitory impact of FOXD2-AS1 knockdown on cell proliferation was reversed by CRABP2 upregulation, as colony formation assays revealed (Fig. 5C, **p < 0.01, ANOVA). The results of TUNEL assays demonstrated that CRABP2 also rescued the decrease in cell apoptosis caused by FOXD2-AS1 deficiency (Fig. 5D-E, **p < 0.01, ***p < 0.001, ANOVA). As wounding healing assays and Transwell assays indicated, CRABP2 elevation promoted OSCC cell migration and invasion in the context of FOXD2-AS1 knockdown (Fig. 5F-I, *p < 0.05, **p < 0.01, ANOVA). The above CCK-8, colony formation, TUNEL, wounding healing and Transwell assays were all repeated in triplicate. In summary, these findings demonstrated that FOXD2-AS1 enhances OSCC malignant cell processes by upregulating CRABP2 expression.

## Discussion

Increasing cancer-related lncRNAs have been found to participate tumor initiation and metastasis in OSCC [30]. Recently, lncRNA ENAH-202 has been reported to aggravate OSCC cell proliferation and metastasis by binding to ZNF502 and promoting the transcription of vimentin [31]. LncRNA DDX59-AS1 is recognized as an oncogenic biomarker in OSCC and is negatively related to immune cells including Mast cell, T cells, B cells, and Treg [32]. FOXD2-AS1 is a tumor-associated gene and is mentioned in various types of cancer [33]. For example, FOXD2-AS1 is upregulated in cervical cancer tissues especially in samples with advanced tumor staging, and it promotes cervical cancer malignant progression by decreasing CDX1 level [34]. FOXD2-AS1 increases KLK7 level by interacting with miR-485-5p, thereby exacerbating cell proliferation and migration in papillary thyroid cancer [35]. A published article analyzed that aberrant FOXD2-AS1 expression can enhance malignant activities of OSCC cells based on TCGA data [36]. Consistent with previous studies, the present work also highlighted the oncogenic role of FOXD2-AS1 in OSCC. However, the current study not only validated the effect of FOXD2-AS1 on cell growth, migration, and invasion but also illustrated the underlying mechanism. We found that FOXD2-AS1 expression was upregulated in OSCC cells and its knockdown repressed OSCC cell proliferation, invasion, and migration while promoting cell apoptosis. LncRNA can act as a sponge to interact with miRNAs and interfere the miRNA functions [30]. The current study identified miR-378 g as the downstream factor of FOXD2-AS1 based on bioinformatics analysis. FOXD2-AS1 interacted with miR-378 g and suppressed its expression, thereby hampering the inhibitory impact of miR-378 g on CRABP2 expression.

MiRNA contains 20–25 nucleotides and post-transcriptionally regulates the expression of target genes, thereby destroying the stability of mRNAs [37]. MiR-378 g acts as a tumor-suppressor in many types of cancer. For example, miR-378 g, as a member of the miR-378 family, is downregulated in colorectal cancer tissues [38]. MiR-378 g mimics promote radiosensitivity, enhance apoptosis of oncogenic cells and inhibit cell invasion in nasopharyngeal carcinoma [39]. In addition, miR-378 g is involved in ceRNA networks during its participation in tumor initiation and development. LINC00888 competes with the gene transferrin receptor (TFRC) for the chance of interacting with miR-378 g and leads to the upregulation of TFRC, thereby accelerating laryngeal cancer cell growth and mobility [40]. MiR-378 g is also mentioned in the HOXC13-AS1/miR-378 g/HOXC13 axis in OSCC [41]. In line with these articles, miR-378 g expression was downregulated in OSCC cells and had the tumor-suppressive potential in this study. In addition, miR-378 g was negatively correlated to FOXD2-AS1 expression or CRABP2 expression. MiR-378 g bound to 3’untranslated region (3’UTR) of CRABP2 at the position of 211–218.

CRABP2, a small intracellular protein, is a member of the intracellular lipid-binding proteins family [42]. It plays an oncogenic or tumor-suppressive role in different types of cancer. For example, CRABP2 inhibits the invasion and metastasis of ER + breast cancer and facilitates the metastasis of ER- breast cancer by regulating the stability of Lats1 [42]. CRABP2 promotes epithelial mesenchymal transition (EMT) in serous ovarian cancer by upregulating the expression of enhancer of zeste homolog 2 and promoting the methylation of tripartite motif containing 16 (TRIM16) [43]. CRABP2 has been reported to be downregulated in human papillomaviruses-positive oropharyngeal squamous cell carcinoma by Martinez in 2007 [44]. In addition, an article published in 2009 revealed that the absence of CRABP2 is closely related to low survival rate of head and neck carcinoma, suggesting the anticancer role of CRABP2 [45]. Different from these findings in relevant articles, CRABP2 in the current study was revealed to be upregulated in OSCC cells and its expression was positively correlated to FOXD2-AS1 expression. In addition, CRABP2 overexpression promotes cell proliferation, migration, and invasion in the context of FOXD2-AS1 knockdown, suggesting that FOXD2-AS1 aggravates malignant behavior of OSCC cells by upregulating CRABP2. Since the role of CRABP2 in oral cancer is controversial, more experiments should be conducted in the future for further exploration. In other types of cancer, CRABP2 is shown to be strongly associated with hippo pathway and integrin/FAK pathway [42, 46–48]. Whether CRABP2 can regulate OSCC malignant behavior by affecting the activation of the two pathways can be further explored in the future.

In conclusion, FOXD2-AS1 is upregulated in OSCC cells, and it enhances malignant cell processes by interacting with miR-378 g and thus elevating CRABP2 expression. FOXD2-AS1, miR-378 g, and CRABP2 expression might be correlated with clinical stages and survival outcome and can become potential diagnostic biomarkers for OSCC. Targeting the FOXD2-AS1/miR-378 g/CRABP2 axis might be a promising strategy for the investigation of target therapy.

To be honest, there are also several limitations in our study. First, animal experiments were not carried out to explore the in vivo role of FOXD2-AS1. In addition, OSCC samples were not collected to measure the expression of FOXD2-AS1, miR-378 g, and CRABP2 in clinical tissues at different stages. Moreover, the experimental results require to be further validated in other OSCC cell lines to avoid potential biases among different cell lines. CRABP2 can also affect EMT process and chemoresistance to cancer cells [49, 50]. The effects of the FOXD2-AS1/miR-378 g/CRABP2 axis on chemosensitivity and EMT were not included in the study but can be the direction for future work.

### Electronic supplementary material

Below is the link to the electronic supplementary material.

Supplementary Material 1

## Data Availability

The datasets used or analyzed during the current study are available from the corresponding author on reasonable request.
