# Supplementary material for: FOXD2-AS1 promotes malignant cell behavior in oral squamous cell carcinoma via the miR-378 g/CRABP2 axis
Source: BMC Oral Health. 2024 May 28;24:625. doi: 10.1186/s12903-024-04388-2 (PMC11134640; doi:10.1186/s12903-024-04388-2)

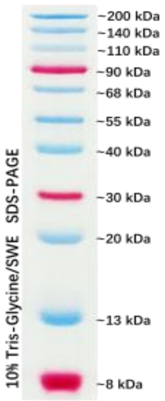

15kDa CRABP2

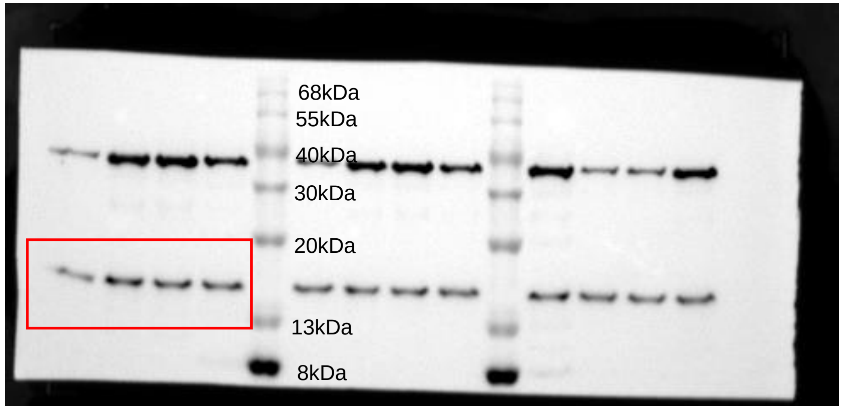

HOK Cal27 SCC9 HSC-3

CRABP2

GAPDH

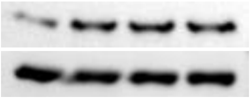

HOK Cal27 SCC9 HSC-3

GAPDH 36kDa

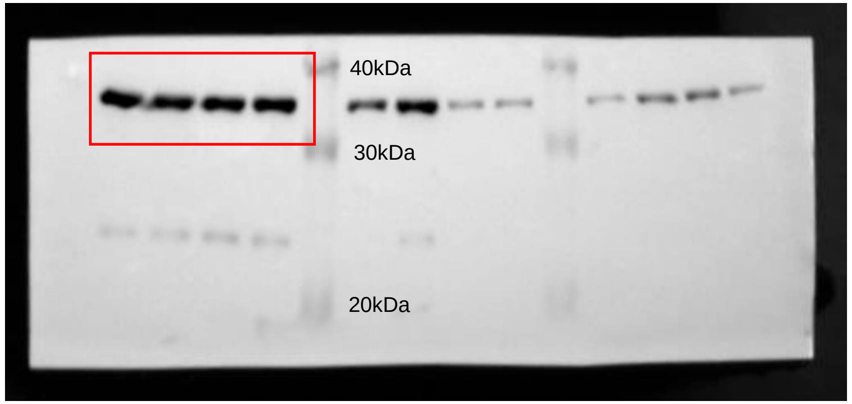

HOK Cal27 SCC9 HSC-3

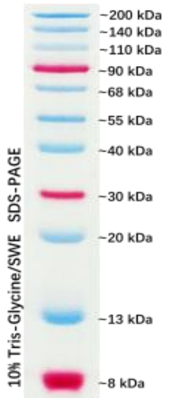

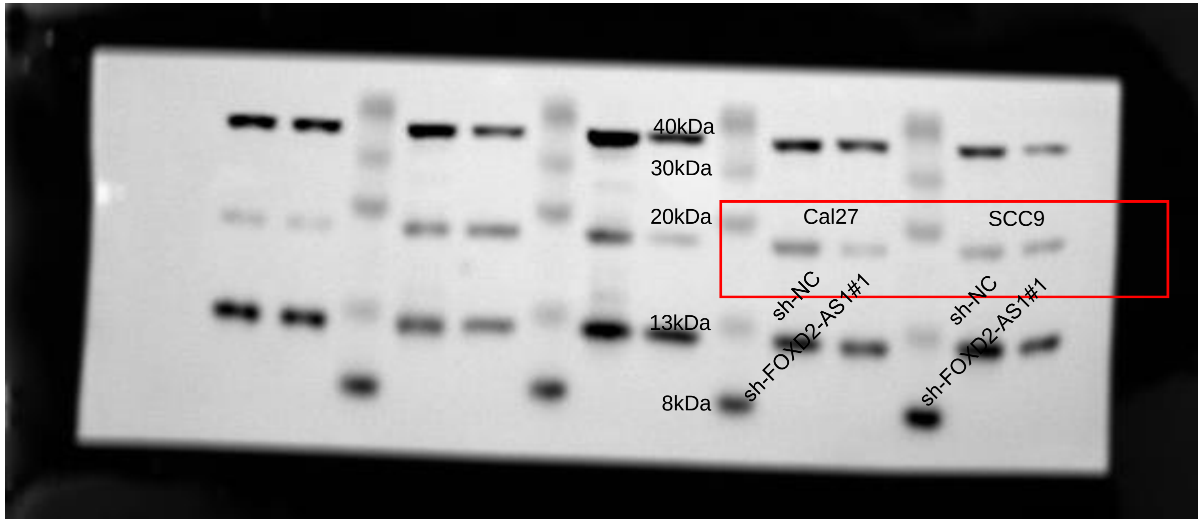

CRABP2 15kDa

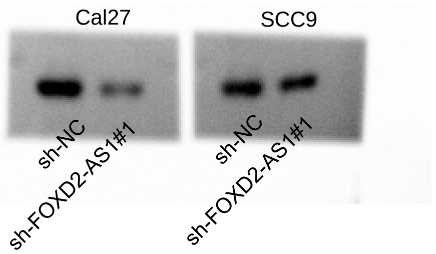

GAPDH 36kDa

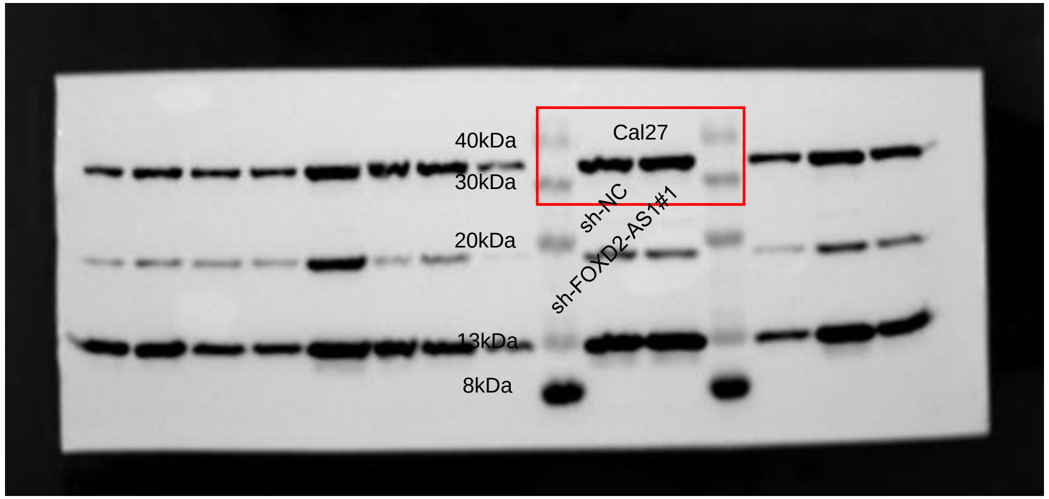

GAPDH 36kDa

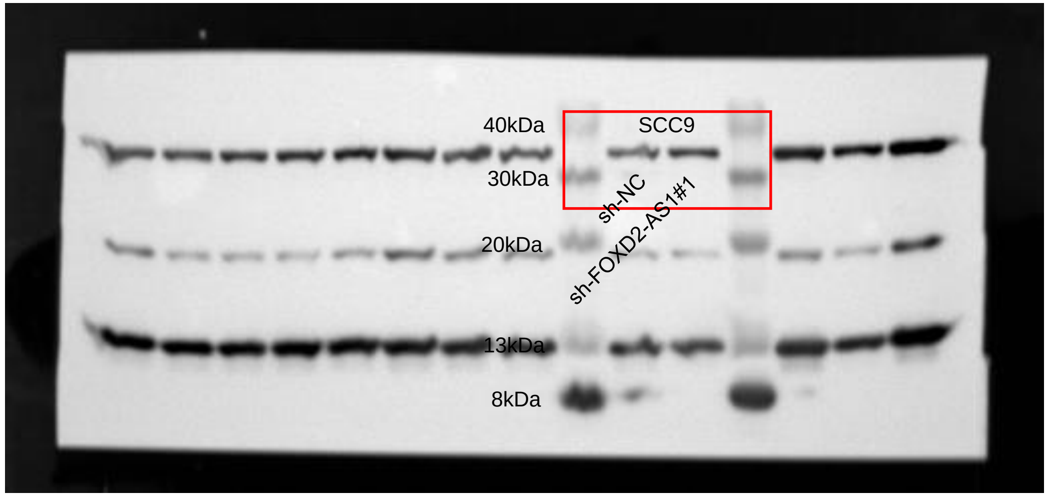

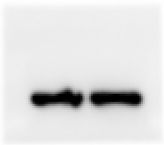

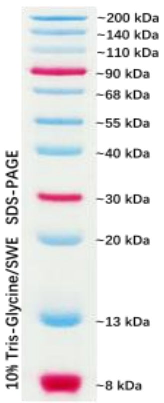

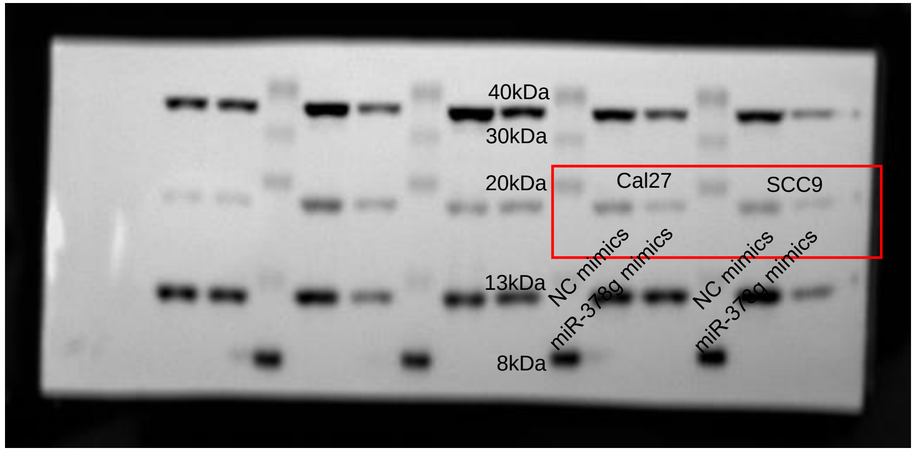

CRABP2 15 kDa

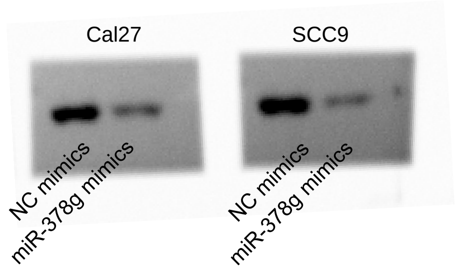

GAPDH 36kDa

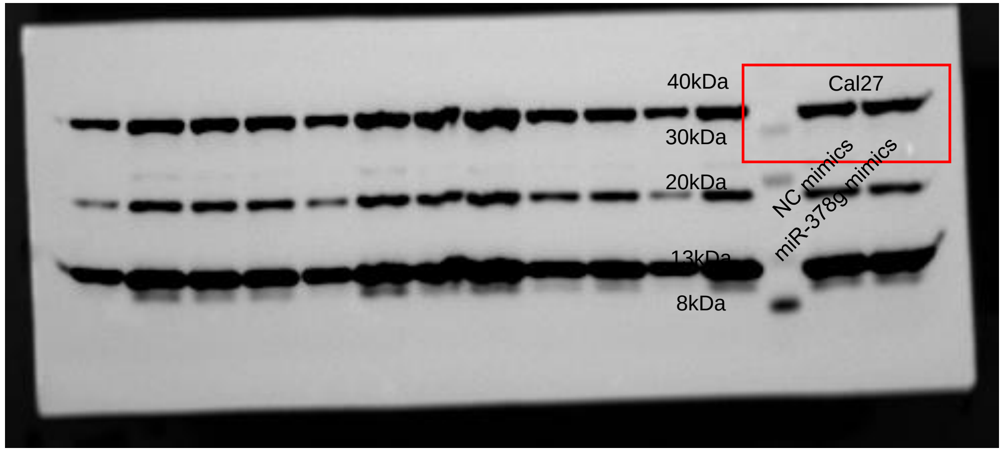

GAPDH 36kDa

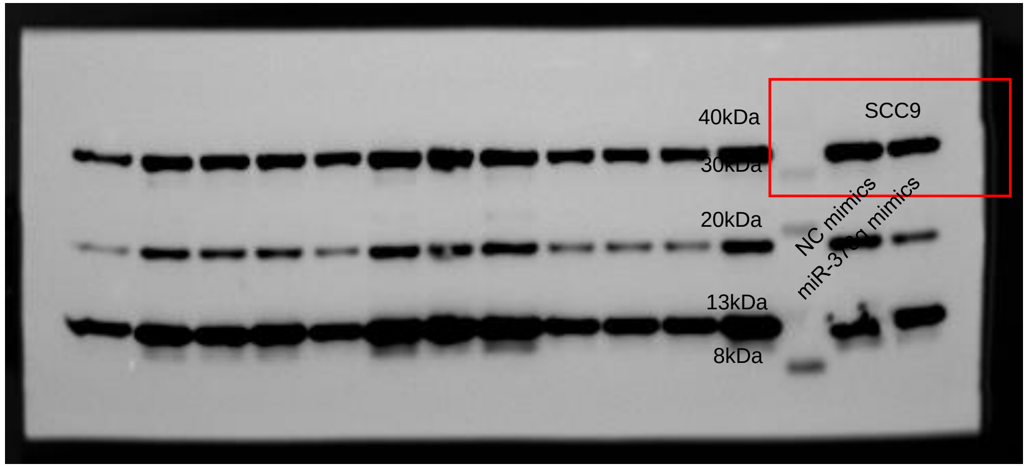

Supplement: Supplementary file 1 — Supplementary Material 1 [file 12903_2024_4388_MOESM1_ESM.pdf]
